# Draft genome sequence analysis of multidrug-resistant *Salmonella enterica* subsp. *enterica* serovar Mbandaka harboring colistin resistance gene *mcr*-*9.1* isolated from foals in Kentucky, USA

**DOI:** 10.1128/mra.00737-24

**Published:** 2024-10-07

**Authors:** Yosra A. Helmy, Ajran Kabir, Mohamed Saleh, Laura A. Kennedy, Logan Burns, Beth Johnson

**Affiliations:** 1 Maxwell H. Gluck Equine Research Center, Department of Veterinary Science, Martin-Gatton College of Agriculture, Food, and Environment, University of Kentucky, Lexington, Kentucky, USA; 2 Veterinary Diagnostic Laboratory, Department of Veterinary Science, Martin-Gatton College of Agriculture, Food, and Environment, University of Kentucky, Lexington, Kentucky, USA; 3 Division of Lab Services, Kentucky Department for Public Health, Frankfort, Kentucky, USA; University of Maryland School of Medicine, Baltimore, Maryland, USA

**Keywords:** multidrug-resistant, *Salmonella*, colistin resistance, thoroughbred foals, Kentucky, *Salmonella *Mbandaka, *mcr*, draft genome

## Abstract

Horses play a significant role in the direct or indirect transmission of *Salmonella* to humans. Here, we report the draft genomes of multidrug-resistant *Salmonella enterica* subsp. *enterica* serovar Mbandaka YAH-F68 isolated from foals in Kentucky, USA belonging to sequence type 413 and harboring the mobile colistin resistance gene *mcr*-*9.1*.

## ANNOUNCEMENT

*Salmonella enterica* is a major foodborne pathogen with a public health impact worldwide (1–3). *Salmonella* Mbandaka has been recognized by the Centers for Disease Control and Prevention as a significant outbreak-causing serotype (4). *Salmonella* Mbandaka ST413 is one of the top 10 *Salmonella* serotypes causing human illnesses in Europe (5). There were no reported outbreaks in equines; however, three sequences from horses in North America are available in GenBank (4). The presence of *mcr-9.1* gene in *Salmonella* reduces its sensitivity to colistin, and colistin-resistant *Salmonella* might compromise the complicated gastroenteritis treatment (6). Here, we present the draft genome of one MDR *Salmonella* Mbandaka YAH-F68 (ST413) harboring *mcr-9.1* gene.

For bacterial isolation, 1 gram of feces was collected by the UK Vetrinary Diagnostic Laboratory from a 4-month-old thoroughbred foal suffering from colitis in central Kentucky, enriched in 9 mL of tetrathionate broth, and incubated at 37°C for 24 h. Enriched samples were subcultured in xylose lysine tergitol 4 media and incubated at 37°C for 24 h. Suspected black colonies were confirmed by PCR following DNA extraction using boiling method (7, 8). For the genomic DNA preparation, the confirmed colony was subcultured in Blood Agar Plates (Hardy Diagnostics, CA, USA) and incubated overnight at 37°C. Genomic DNA was extracted using Qiagen DNeasy Kit (Qiagen, MD, USA) on QIAcube Connect instrument (Qiagen, MD, USA). The genomic library was prepared using the Illumina DNA Prep Kit (Illumina, CA, USA). Genome sequencing was conducted on MiSeq Illumina platform generating 2 × 300 bp-long paired-end reads. Adapter trimming from paired-end reads (*n* = 1,442,773) was performed using Trimmomatic version 0.40 (9), and *de novo* assembly was performed on SPAdes version 4.0 (10). The quality of the raw reads was evaluated using FastQC version 0.12.1 (11). Assembled contigs were annotated using PGAP version 6.7 (NCBI Prokaryotic Genome Annotation Pipeline) (12). Sequence typing was performed on mlst version 2.23.0 (https://github.com/tseemann/mlst). Resistance genes and plasmids were detected on ABRicate version 1.0.1 (https://github.com/tseemann/abricate) using the NCBI database and PlasmidFinder database (https://bitbucket.org/genomicepidemiology/plasmidfinder_db/src/master/), respectively. Virulence factor genes were identified using VFanalyzer (13). Unless otherwise noted, all the aforementioned software was run with its default settings.

The assembled *Salmonella* Mbandaka YAH-F68 strain comprised 79 contigs (*N*_50_: 254,896 bp) with a GC content of 51.96%. The assembled genome length was 5,054,112 bp with a genome coverage of 120×. Based on PGAP analysis, this genome had a total of 4,977 genes, 4,883 coding sequences, including 94 RNA genes, 2 CRISPR arrays, 14 mobile genetic elements including 3 plasmids (IncHI2A_1, RepA_1_pKPC-CAV1321, and IncHI2_1), and five prophage regions including 116 prophage genes. The genome was typed as ST413 based on the pubMLST database. The presence of the *mcr*-*9.1* gene was detected in contig-41, and the contig showed 100% similarity to the corresponding region in plasmid p628-mcr (CP091551.1) from *Salmonella* and pFF2-1h-mcr-9 (CP097199.1) from *Escherichia coli*. Interestingly, other resistance genes were detected, including *sul2, aac(6′)-llc, aac (3)-ll, arr-2, ere(A), bla_SHV-12_, aph(3′)-la, dfrA19, aph (6)-ld, aph(3″)-ld, aac(6′)-lb, catA2, sul1*, and *aadA2*, which are responsible for phenotypic resistance to sulfonamides, aminoglycosides, rifampin, macrolides, cephalosporins, trimethoprim, streptomycin, and chloramphenicol. Furthermore, 127 virulence factor genes were predicted in this genome.

## Data Availability

The WGS of *Salmonella* Mbandaka YAH-F68 strain was deposited to GenBank under the accession number JBEVAA000000000 (BioProject PRJNA1086499, BioSample SAMN42043563). The Illumina raw reads have been deposited at the Sequence Read Archive (SRA) under accession number SRS21752683.
